# Pilot randomized controlled trial of an internet-based smoking cessation intervention for pregnant smokers (‘MumsQuit’)^☆^

**DOI:** 10.1016/j.drugalcdep.2014.04.010

**Published:** 2014-07-01

**Authors:** Aleksandra Herbec, Jamie Brown, Ildiko Tombor, Susan Michie, Robert West

**Affiliations:** a Cancer Research UK Health Behaviour Research Centre, Department of Epidemiology and Public Health, University College London, 1-19 Torrington Place, London WC1E 7HB, UK; b Department of Clinical, Educational and Health Psychology, University College London, 1-19 Torrington Place, London WC1E 7HB, UK; c National Centre for Smoking Cessation and Training, 1–6 Yarmouth Place, London W1J 7BU, UK

**Keywords:** Smoking cessation, Pregnancy, Internet, Self-help

## Abstract

**Background:**

Internet-based Smoking Cessation Interventions could help pregnant women quit smoking, especially those who do not wish to, or cannot, access face-to-face or telephone support. This study aimed to preliminarily evaluate the effectiveness and usage of a fully automated smoking cessation website targeted to pregnancy, ‘MumsQuit’, and obtain an initial effect-size estimate for a full scale trial.

**Methods:**

We recruited 200 UK-based pregnant adult smokers online to a two-arm double-blind pilot RCT assessing the effectiveness of MumsQuit compared with an information-only website. MumsQuit was adapted from a generic internet smoking cessation intervention, ‘StopAdvisor’. The primary outcome was self-reported continuous 4-week abstinence assessed at 8 weeks post-baseline. Secondary outcomes were automatically collected data on intervention usage.

**Results:**

Participants smoked 15 cigarettes per day on average, 73% were in the first trimester of their pregnancy, 48% were from lower socioeconomic backgrounds, and 43% had never used evidence-based cessation support. The point estimate of odds ratio for the primary outcome was 1.5 (95% CI = 0.8–2.9; 28% vs. 21%). Compared with control participants, those in the MumsQuit group logged in more often (3.5 vs. 1.3, *p* < 0.001), viewed more pages (67.4 vs. 5.7, *p* < 0.001) and spent more time browsing the website (21.3 min vs. 1.0 min, *p* < 0.001).

**Conclusions:**

MumsQuit is an engaging and potentially helpful form of support for pregnant women who seek cessation support online, and merits further development and evaluation in a full-scale RCT.

## 1 Introduction

Smoking in pregnancy is damaging to fetal and maternal health (Einarson and Riordan, 2009; Hackshaw et al., 2011). In countries such as the UK and US it is estimated that between 10% and 20% of pregnant women smoke throughout the pregnancy (Health and Social Care Information Centre, 2012; Jones et al., 2012; Tong et al., 2013). Prevalence is higher among younger women and those from lower socio-economic status (SES; Graham et al., 2010). Most pregnant smokers report that they want to quit (Ussher et al., 2004), but only approximately half succeed in stopping before giving birth (Health and Social Care Information Centre, 2012).

Face to face behavioral support aids smoking cessation in pregnancy (Chamberlain et al., 2013). In the UK it is mainly offered through National Health Services (NHS) Stop Smoking Services, which include dedicated services for pregnant women. These are universally available and free at the point of access, and 47% of pregnant attendees who set a quit date with the services achieve 4-week biochemically verified abstinence (Health and Social Care Information Centre, 2013). However, the uptake of face-to-face cessation support remains as low as 5% among pregnant smokers (Jones et al., 2012). A number of barriers reported by pregnant women may contribute to this, including limited service availability, time constrains (Ussher et al., 2006), as well as negative expectations and fear of stigmatization and disappointment in case of failure (Bryce et al., 2009).

More traditional forms of self-help cessation aids, such as booklets or text-messaging, may be acceptable for pregnant smokers (Naughton et al., 2013; Ussher et al., 2004), but the evidence of their effectiveness is inconsistent (Naughton et al., 2012, 2008; Pollak et al., 2013). Moreover, the vast majority of UK quit attempts remain completely unaided (Raupach et al., 2013). There is therefore a need to develop new and complementary methods of delivering effective evidence-based cessation support for pregnant smokers.

The internet offers the prospect of attractive and highly cost-effective means of delivering behavioral cessation support, particularly in countries such as England, where 70% of smokers in the general population have access to internet (Brown et al., 2013). Internet-based smoking cessation interventions may be suitable for pregnant smokers, as they can offer non-judgmental and flexible assistance that is valued by pregnant women (Butterworth et al., 2013), and additionally one that does not require them to engage with smoking cessation services or health professionals face-to-face (Tombor et al, 2014). Online interventions can also offer readily available treatment, as well as close and ongoing monitoring of behavior and progress that may be especially helpful in smoking cessation treatment for pregnant women (Heil et al., 2009). Finally, smoking cessation websites can also deliver more comprehensive, tailored and interactive support than other forms of self-help, which in turn can increase their relevance (Te Poel et al., 2009) and effectiveness compared with booklets or e-mail-based interventions (Shahab and McEwen, 2009).

To the best of our knowledge no studies have been published on smoking cessation websites that were specifically designed for pregnant smokers, and we lack data on usage and potential effectiveness of such intervention in this population of smokers. We aimed to address this gap by evaluating a novel online intervention targeted to quitting smoking in pregnancy (‘MumsQuit’), which was adapted from ‘StopAdvisor’, a generic website for adult smokers (a detailed description of the intervention flow see Brown et al., 2012; Michie et al., 2012). The development of StopAdvisor was informed by 19 theoretical propositions identified from the PRIME theory of motivation and addiction (West and Brown, 2013), 33 evidence- or theory-based behavior change techniques, 26 web-design principles and nine principles from user-testing with lower SES smokers (Michie et al., 2012).

The overarching theme of both StopAdvisor and MumsQuit is to emulate sessions with an NHS smoking cessation expert who provides ongoing support, offers a structured quit plan and personalizes the advice for the users. This is achieved through interactive menus that allow participants to explore answers to common questions, facts, videos and testimonials of ex-smokers on smoking, cessation and addiction; multimedia features supporting cravings (e.g., relaxing music and meditation exercises); as well as tunneled sessions, which direct the users through a series of pages over which they have little navigational control. The use of a tunneled architecture allows the website to systematically gather information while simulating a dialog with a ‘StopAdvisor’ who provides tailored feedback and advice relating to quit date, smoking status, use of medication, current cravings levels, confidence in quitting, and anticipated stressful or social situations.

The adaptation of MumsQuit to pregnancy was limited to changes in the design (e.g., inclusion of imagery appealing to the identities of a pregnant ex-smokers) and adjustment of some of the content (e.g., adjustment of information on cessation medication use in pregnancy) to focus on pregnancy. Although these changes are minimal, research shows that targeting of interventions to shared characteristics of user groups can increase their relevance and reach (Kreuter and Skinner, 2000; Strecher, 1999). Such targeting has been previously successfully used in self-help smoking cessation treatments for adolescents (Meis et al., 2002), elderly (Rimer et al., 1994), and ethnic minorities (Swartz et al., 2006; Windsor et al., 1985). Moreover, research on development of other health interventions targeted to pregnancy, such as for management of diabetes, were shown to be positively received by pregnant women (Adolfsson and Jansson, 2012; Lie et al., 2013). Therefore, offering a targeted to pregnancy version of an internet-based smoking cessation intervention might be crucial for engaging this population in use of evidence-based support with quitting.

In line with the Medical Research Council recommendations on development and evaluation of complex interventions (Craig et al., 2008), the present study aimed to obtain a preliminary indication of the likely effectiveness of MumsQuit relative to an information-only website and to obtain information on usage patterns that could inform development of a version that would be suitable for evaluation in a full-scale randomized controlled trial (RCT).

## 2 Methods

### 2.1 Design

Participants were recruited to a two-arm double-blind pilot RCT. We compared the effectiveness of a fully automated ‘MumsQuit’ intervention with an information-only control website at 8 weeks follow-up. Each condition was delivered online with no face-to-face contact. The study was approved by the UCL Research Ethics Committee (3556/002).

### 2.2 Participants

We aimed to recruit 200 participants. This was a pragmatic choice based on expected recruitment rate (Park et al., 2007; Pollak et al., 2013) and the aim of establishing a point estimate of effect size that could be used when designing a full-scale trial. This sample size had 42% power to detect a difference in success rates of 25% in the active condition vs. 15% in the control, which is the estimated 4-week quit rate from unaided quitting in the general population (25). The rate of 25% was based on the pilot study of StopAdvisor (Brown et al., 2012).

Recruitment was conducted between March, 2012 and October, 2013 through an online advertisement placed on NHS Smokefree website devoted to smoking cessation in pregnancy, as well as discussion forums and websites for UK pregnant women. The recruitment materials emphasized that the trial was of a new intervention developed at University College London, UK, and which was targeted to pregnancy and did not involve face-to-face contact. Inclusion criteria for the trial were: having internet access, being female, pregnant, aged 18 or more, UK-based, smoking daily, willing to make a serious quit attempt, and use a stop-smoking website which sends email reminders, as well as agree to be followed up over the telephone at 2 months, and provide informed consent. Participants were not compensated for participation in the study, nor for completing follow-up assessments.

### 2.3 Measurements

We collected the following data on characteristics of participants at baseline (presented in Table 1): pregnancy trimester (first, second, third); socio-demographic characteristics (age; marital status; having children already, ethnicity, education level and current educational status); socioeconomic status (SES), with ‘lower’ identified as those in long-term employment, or working in manual and routine settings; smoking characteristics (cigarettes smoked per day, age of smoking initiation, nicotine dependence measured by the Heaviness of Smoking Index (HSI) (Kozlowski et al., 1994)), and the Fagerström Test for Nicotine Dependence (Heatherton et al., 1991), time spent with urges to smoke and their strength in the past 24 h (Ussher et al., 2013), as well as confidence in quitting (on a scale from 1 to 7); Mood and physical symptoms scale (MPSS; West and Hajek, 2004); and finally, history of quit attempts (cessation support accessed previously, length of the longest quit attempt). We also automatically collected data on the initial level of engagement with the study website (number of recruitment pages viewed and time spent on the website before providing consent).

The primary outcome was self-reported 4-week continuous abstinence at 8-week follow-up. Secondary outcomes included automatically collected data on quantifiable website usage (number of log ins, number of pages viewed, and time spent browsing the website), excluding the recruitment period.

### 2.4 Intervention

MumsQuit offers an interactive, personalized, and structured quit plan that emulates the support from an expert smoking cessation advisor from NHS Stop Smoking Services. The intervention delivers 33 evidence- or theory-based behavior change techniques (for details on StopAdvisor see Brown et al., 2012; Michie et al., 2012, 2013) and provides up to 4 weeks of pre-quit date support and up to 4 weeks of post-quit date support, with e-mail reminders sent to notify users when new intervention sessions are being released.

The development of MumsQuit involved the following specific adaptation of the content and design of StopAdvisor to pregnancy: removing Varenicline and Bupropion as medication options; qualifying advice on Nicotine Replacement Therapy; adding information about risks of maternal smoking to the fetus and benefits of quitting in pregnancy; adding imagery appealing to the identities of an ex-smoking pregnant women and a mother, and replacing an avatar of ‘generic’ female cessation advisor in StopAdvisor with one resembling a female midwife or a doctor.

The control condition involved a one-page static, non-personalized website that provided brief standard advice for users. The content of the control website was based on a widely used manual for smoking cessation support for practitioners (McEwen et al., 2008). Both MumsQuit and the control website were developed using a free and open-source software, LifeGuide (Hare et al., 2009), which was specifically created to help researchers develop and evaluate Internet-based behavior change interventions. No changes were made to MumsQuit or the control condition during the trial. For screenshots of MumsQuit and the control website see Supplementary Materials.^1^ Flowcharts of the intervention structure are published elsewhere (Michie et al., 2013).

### 2.5 Procedures

Recruitment was conducted online, and participants were contacted by the research team only at follow-up. Before providing consent, participants read the study information pages that outlined the procedures, inclusion criteria, and they were encouraged to contact the authors in case of any questions or comments. After providing consent, participants completed the baseline assessment, which included the provision of their contact details. After this, they were randomized by the computer to receive access to either MumsQuit or the control condition, with allocation concealment and locking of e-mails to minimize duplicate sign ups. Consenting participants were screened by a researcher before being included in the trial: participants who did not meet the inclusion criteria (e.g., due to being male or not currently pregnant) were allowed to use the websites but were not included in the trial (*n* = 11).

At 8 weeks post baseline completion, participants were automatically followed up through e-mail and asked to complete an online self-assessment questionnaire on primary outcome and secondary outcomes. Participants who did not complete the online follow-up were contacted over telephone (up to five telephone contact attempts) by AH and IT, who were blind to intervention allocation, to gather information only on the primary outcome. No technical issues interrupted the study.

### 2.6 Analysis

In the primary analysis, abstinence rates from MumsQuit and the control condition were compared using logistic regression according to the intention-to-treat (ITT) principle with participants lost to follow-up treated as smokers. In a secondary analysis, the two conditions were also compared using a logistic regression model adjusting for all baseline characteristics. In a further secondary analysis, the abstinence rates of complete cases were compared to assess how attrition has affected the results. Data on satisfaction assessment were analyzed using logistic regression. For logistic regression analyses the associated odds ratio and 95% confidence interval were calculated. Data on website usage were compared across conditions using an independent *t*-test, without the assumption of equality of variance where a significant difference in variance was found between the two arms. The use of additional support was analyzed using chi-square tests with Fisher's exact tests. Alpha for the primary and secondary outcomes was set to 0.05, and was adjusted using Sidak criterion for multiple comparisons of baseline characteristics.

## 3 Results

**Fig. 1:**
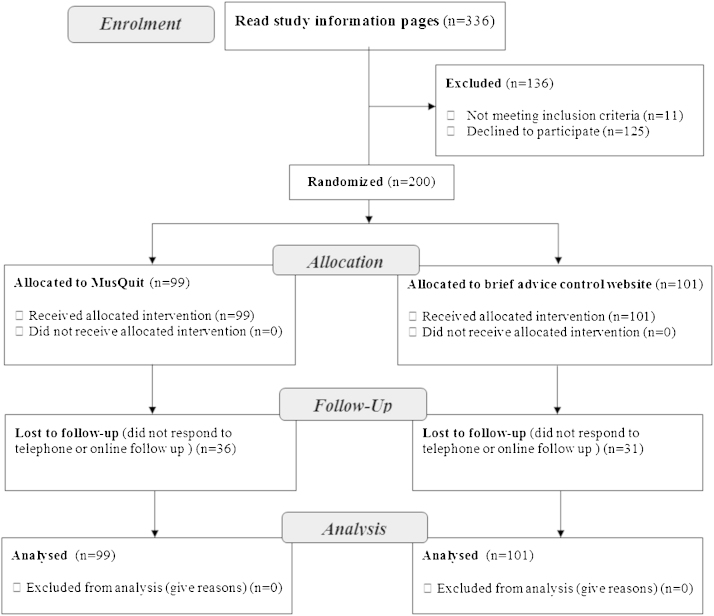
Flowchart of participants.

Fig. 1 shows the flowchart of participants. During the recruitment period, 336 people accessed the information pages of the study website, and 200 (59.5%) consented to participate, of which 99 were randomly allocated to MumsQuit and 101 to the control condition. Of those, 67 (33.5%) were lost to follow up at 8 weeks, with no significant differences between MumsQuit and control (36.4% vs. 30.7%, *p* = 0.40). Participants’ characteristics are presented in Table 1. Participants smoked approximately 15 cigarettes per day, 73% were in the first pregnancy trimester, 48% were from ‘lower’ SES (those who never worked, were long term unemployed or were from routine and manual occupations), and 43% had never used evidence-based cessation support. There were no significant differences between the RCT arms on any of the baseline characteristics, including the level of engagement with the recruitment webpages at the time of signing up to the study.

A difference of 8% was found in the 4-week continuous abstinent rates between MumsQuit (28.3%) and the control (20.8%); the odds ratio was 1.5 (95% CI = 0.8–2.9) (see Table 2). The results did not change after adjusting for baseline characteristics or in the complete case analysis. There was no indication that the telephone follow-up inflated abstinence rate, as participants followed up by telephone reported still smoking more often than those responding online (69.2% vs. 50.0%). MumsQuit participants logged in significantly more often (3.5 vs. 1.3, *p* < 0.001), viewed more pages (67.4 vs. 5.7, *p* < 0.001) and spent longer time with browsing the website (21.3 min vs. 1.0 min, *p* < 0.001) (see Table 3).

## 4 Discussion

The study showed that an internet-based smoking cessation interventions targeted to pregnancy was a potentially useful cessation aid for pregnant adult smokers. The usage patterns indicated that MumsQuit was engaging. The point-estimate for use in future trials of the effect size in terms of odds ratio was 1.5.

We found that pregnant smokers with either low or high SES may be interested in using online interventions to help them quit smoking, which could help closing the gap between social inequalities in the access to evidence-based cessation treatments (Murray et al., 2009). This corroborates findings from the general population showing that smokers across SES spectrum express interest in internet-based support (Brown et al., 2013). Moreover, internet may offer a viable way of engaging pregnant women who have not previously accessed evidence-based support and are at the early stages of their pregnancy, when quitting may confer the greatest benefit (Bickerstaff et al., 2012). In addition, pregnant smokers who are interested in using online interventions may represent a more dependent group of UK smokers, as suggested by the number of cigarettes smoked per day (Fidler et al., 2011). This is common among treatment-seeking smokers, but it also suggests that these women may require even more intensive support from a smoking cessation website.

The recruitment rate for the present study was low, requiring 18 months to reach the target sample size. Future studies should assess the demand for internet support among this population, and consider recruitment through clinical settings. However, this is also known to be challenging (Naughton et al., 2012; Park et al., 2007; Pollak et al., 2006), and may lead to underrepresentation of women who do not access care from health professionals. Furthermore, the low recruitment rate may be due to a relatively small pool of potential participants in any given year – approximately 85,000 pregnant women in the UK annually (Health and Social Care Information Centre, 2012), and a very narrow time window to recruit them. Therefore, it may be difficult to conduct a large enough traditional RCT of such an intervention among pregnant women that would be able to detect the medium effect size expected. This important challenge may be addressed by international collaboration, allowing for simultaneous evaluation of the intervention in comparable settings in several countries.

Future evaluations of smoking cessation websites targeted to pregnancy may also benefit from a variety of experimental designs. For example adoption of multiphase optimization strategy (MOST) and the sequential multiple assignment randomized trial (SMART) designs (Collins et al., 2007) can help to tease out the relative efficacy of components within an intervention and promote intelligent use of information and resources within a trial. These, together with adoption of shorter follow up times might help to address the issues arising from attrition in Internet-based studies and provide valuable data for rapid or ‘online’ intervention optimization in the transient population of pregnant smokers.

Both MumsQuit and the control websites were based on guidelines for smoking cessation treatment delivered in the UK, which encourage complete cessation and ‘not a puff’ rule following the quit date (Shahab and Kenyon, 2013). Conscious of the assessment burden during pregnancy, we therefore prioritized assessment to align with our intervention: we collected data only on abstinence rates, and not on smoking reduction. However, future studies should also collect information on cutting down as valuable process data and an indication of the intervention's potential effectiveness for smokers who may not be able to quit smoking completely, but who could nevertheless cut down on smoking substantially.

The present study had several limitations. We relied on self-reported data on abstinence, so the observed quit rates are likely to be an overestimation. However, the risk of differential reporting between the conditions is less than for traditional RCTs, in which participants are recruited in person. A related issue is that the reporting of smoking status at the follow-up could have been biased by treatment allocation. For example, it is possible that those receiving MumsQuit realized they had been allocated to the relatively intense support and may have under-reported smoking to a greater extent than control participants. We did not explicitly assess beliefs about allocation but recommend such assessment in future trials. Nevertheless, we believe the risk for this happening in the present study was low: the recruitment materials and study information pages did not describe the trial as comparing a superior and an inferior website, nor did they provide any specific information about the content and nature of support provided by each website.

Participants were also followed-up only at short-term, but since most relapses happen within 4 week from a quit date, the present quit rates are an informative indicator of long-term success (West and Stapleton, 2008). Additionally, due to attrition from online follow-up we were unable to comprehensively assess satisfaction with the website and use of additional support among trial participants. Future studies should embed within the intervention ongoing evaluation of the website to capture data from a larger proportion of users before they become lost to follow up.

Generalizability of the findings is further limited by the self-selection resulting from online-only recruitment, with the sample likely to be more motivated than those in the wider population. However, the key advantage of online interventions is that they may appeal to and reach women who otherwise would not be seeking professional assistance through established services. This is suggested by a high percentage of participants not having previously accessed evidence-based support. Finally, recruitment was restricted to adult women, and the findings may not apply to pregnant adolescents, who have a smoking prevalence of 35% (Health and Social Care Information Centre, 2012). There is, therefore, a need to involve more diverse population, particularly younger women, in future evaluations of smoking cessation websites.

The present findings warrant conducting a larger trial of MumsQuit intervention that would be sufficiently powered to detect both medium effects in the primary outcomes, as well as differences in secondary outcomes. Future work should also focus on identifying better ways of recruiting and retaining pregnant smokers in studies of online interventions. Finally, we still lack studies assessing the demand for website support among pregnant smokers in different countries, and how such interventions could best contribute to wider cessation efforts in this population and traditional support.

## Role of funding source

AH is funded by British Heart Foundation PhD Studentship. The project is funded by a grant from the National Prevention Research Initiative (G0802035). The Funding Partners relevant to this award are (in alphabetical order): Alzheimer's Research Trust; Alzheimer's Society; Biotechnology and Biological Sciences Research Council; British Heart Foundation; Cancer Research UK; Chief Scientist Office, Scottish Government Health Directorate; Department of Health; Diabetes UK; Economic and Social Research Council; Engineering and Physical Sciences Research Council; Health & Social Care Research & Development Office for Northern Ireland; Medical Research Council; The Stroke Association; Welsh Assembly Government. The funding bodies had no role in study design; in the collection, analysis and interpretation of data; in the writing of the report; or in the decision to submit the paper for publication.

## Contributors

All authors contributed to the design of the study. AH, JB, IT and RW contributed to the final content and layout of MumsQuit. AH conducted literature review, developed MumsQuit (through targeting StopAdvisor intervention to pregnancy), was responsible for study advertisement, participant recruitment and follow ups. IT assisted with telephone follow ups. JB managed the data as well as MumsQuit and the control website through LifeGuide software. AH conducted data analysis and wrote the first draft of the manuscript. All authors contributed to and have approved the final manuscript.

## Conflict of interest

JB has received unrestricted research funding from Pfizer. RW undertakes research and consultancy and receives fees for speaking from companies that develop and manufacture smoking cessation medications (Pfizer, J&J, McNeil, GSK, Nabi, Novartis, and Sanofi-Aventis). He also undertakes training for smoking cessation advisors and has a share of a patent for a novel nicotine delivery device. AH, IT and SM have no conflicts of interests.

## Figures and Tables

**Table 1:** Characteristics of the study participants.

Characteristics	MumsQuit (*N* = 99)	Brief-advice control website (*N* = 101)	Total (*N* = 200)
Age in years, mean (SD)	27.6 (6.0)	28.1 (5.8)	27.8 (5.9)
Trimester			
1st trimester, % (*N*)	76.8 (76)	68.3 (69)	72.5 (145)
2nd trimester, % (*N*)	15.2 (15)	22.8 (23)	19.0 (38)
3rd trimester, % (*N*)	8.1 (8)	8.9 (9)	8.5 (17)
Married, % (*N*)	75.8 (75)	77.2 (78)	76.5 (153)
Children, % (*N*)	40.4 (40)	42.6 (43)	41.5 (83)
White ethnicity, % (*N*)	93.9 (93)	91.1 (92)	92.5 (185)
Currently in full-time education, % (*N*)	3.0 (3)	5.0 (5)	4.0 (8)
Had post-16 educational qualifications, % (*N*)	56.6 (56)	62.4 (63)	59.5 (119)
‘Lower’ SES^b^, % (*N*)	54.5 (54)	41.6 (42)	48.0 (96)
Cigarettes per day, mean (SD)	14.6 (5.2)	14.7 (7.8)	14.7 (6.6)
Age of smoking initiation^a^, mean (SD)	15.6 (2.8)	16.1 (2.6)	15.9 (2.7)
Never previously used support in quit attempt, % (*N*)	42.4 (42)	43.6 (44)	43.0 (86)
Quit attempt in previous year, % (*N*)	62.6 (62)	54.5 (55)	41.5 (83)
Confidence in stopping (1–7), mean (SD)	4.7 (1.8)	4.6 (1.6)	4.7 (1.7)
Never stopped for more than a week, % (*N*)	49.5 (49)	55.4 (56)	47.5 (95)
Smoking within 5 min of waking, % (*N*)	40.4 (73)	32.7 (33)	36.5 (73)
HSI (range: 0–6)^b^, mean (SD)	2.7 (1.4)	2.7 (1.4)	2.8 (1.2)
FTND (range: 0–10)^c^, mean (SD)	4.6 (1.7)	4.3 (1.6)	4.4 (1.6)
Time with urges (range: 0–5), mean (SD)	3.0 (1.0)	3.0 (1.1)	3.0 (1.0)
Strength with urges (range: 0–5), mean (SD)	3.2 (0.9)	3.2 (1.1)	3.2 (0.9)
MPSS-mood subscale (0–4)^d^, mean (SD)	2.7 (0.9)	2.7 (0.8)	2.7 (0.9)
Time (min) to complete online recruitment, mean (SD)	9.3 (4.9)	9.9 (85.6)	9.6 (5.2)
Pages viewed to complete online recruitment, mean (SD)	19.3 (1.7)	19.3 (1.4)	19.3 (1.5)

a Data on age of smoking initiation were missing for 1 participant in the control condition.

b Heaviness of smoking index [27].

c Fagerström Test for Nicotine Dependence [28].

d This scale is the mean of responses to five separate questions on: depressed, irritable, restless, hungry and poor concentration.

**Table 2:** Effects of MumsQuit on self-reported 4-week continuous abstinence rates.

	MumsQuit	Brief advice control website	Odds ratio (95% CI)	Exact *p*-value	Percentage-point difference (95% CI)
	*Percent (number)*				
Primary outcome: abstinence at 4 weeks^ITT^	28.3 (28/99)	20.8 (21/101)	1.5 (0.8–2.9) 1.5 (0.7–3.4)^a^	0.220 0.315	7.5 (−4.4 to 19.4)
Secondary outcome: abstinence at 4 weeks^CC^	44.4 (28/63)	30.0 (21/70)	1.9 (0.9–3.8) 2.7 (1.0–7.1)^a^	0.915 0.048	14.4 (−1.9 to 30.8)

^ITT^ intention-to-treat analysis. ^CC^ complete case analysis. ^a^ Adjustment for all baseline characteristics presented in Table 1; one participant was missing data on age of smoking initiation.

**Table 3:** Website usage.

	MumsQuit (*N* = 99)	Brief advice control website (*N* = 101)	*t*-Test	*p*-Value	Mean difference (95% CI)
Website usage	*Mean (SD)*				
Log-ins	3.5 (4.5)	1.3 (0.5)	*t*(100.6) = 4.9	<0.001	2.3 (1.4–3.2)
Total time (min)^a^	21.3 (37.7)	1.0 (2.5)	*t*(98.9) = 5.3	<0.001	20.3 (12.8–27.9)
Total page views	67.4 (92.2)	5.7 (4.0)	*t*(98.4) = 6.7	<0.001	61.7 (43.3–80.1)

a Time on website is an underestimate – time on last page is always unknown in LifeGuide interventions. Interaction between a browser and LifeGuide server happens when a page is loaded. After that, there is no further communication until another page is loaded from the same server. Time on page is calculated by taking the exact time a page was loaded (from the Lifeguide server) and comparing it with the exact time that the previous page in the session was loaded. When a user closes the MumsQuit tab, or just types a different website in to the address bar, no interaction happens between the browser and the server, so it is not possible to identify the time the final action occurred.

## Footnotes

1 Supplementary material can be found by accessing the online version of this paper. See Appendix A for more details.

## References

[bib0005] Adolfsson A., Jansson M. (2012). Prototype for Internet support of pregnant women and mothers with type 1 diabetes: focus group testing. Psychol. Res. Behav. Manag..

[bib0010] Bickerstaff M., Beckmann M., Gibbons K., Flenady V. (2012). Recent cessation of smoking and its effect on pregnancy outcomes. Aust. N. Z. J. Obstet. Gynaecol..

[bib0015] Brown J., Michie S., Geraghty A.W., Miller S., Yardley L., Gardner B., Shahab L., Stapleton J.A., West R. (2012). A pilot study of StopAdvisor: a theory-based interactive internet-based smoking cessation intervention aimed across the social spectrum. Addict. Behav..

[bib0020] Brown J., Michie S., Raupach T., West R. (2013). Prevalence and characteristics of smokers interested in internet-based smoking cessation interventions: cross-sectional findings from a national household survey. J. Med. Internet Res..

[bib0025] Bryce A., Butler C., Gnich W., Sheehy C., Tappin D.M. (2009). CATCH: development of a home-based midwifery intervention to support young pregnant smokers to quit. Midwifery.

[bib0030] Butterworth S.J., Sparkes E., Trout A., Brown K. (2013). Pregnant smokers’ perceptions of specialist smoking cessation services. J. Smok. Cessat..

[bib0035] Chamberlain C., O’Mara-Eves A., Oliver S., Caird J.R., Perlen S.M., Eades S.J., Thomas J. (2013). Psychosocial interventions for supporting women to stop smoking in pregnancy. Cochrane Database Syst. Rev..

[bib0040] Collins L.M., Murphy S.A., Strecher V. (2007). The multiphase optimization strategy (MOST) and the sequential multiple assignment randomized trial (SMART): new methods for more potent eHealth interventions. Am. J. Prev. Med..

[bib0045] Craig P., Dieppe P., Macintyre S., Michie S., Nazareth I., Petticrew M. (2008). Developing and evaluating complex interventions: the new Medical Research Council guidance. BMJ.

[bib0050] Einarson A., Riordan S. (2009). Smoking in pregnancy and lactation: a review of risks and cessation strategies. Eur. J. Clin. Pharmacol..

[bib0055] Fidler J.A., Shahab L., West R., Jarvis M.J., McEwen A., Stapleton J.A., Vangeli E., West R. (2011). ‘The smoking toolkit study’: a national study of smoking and smoking cessation in England. BMC Public Health.

[bib0060] Graham H., Hawkins S.S., Law C. (2010). Lifecourse influences on women's smoking before, during and after pregnancy. Soc. Sci. Med..

[bib0065] Hackshaw A., Rodeck C., Boniface S. (2011). Maternal smoking in pregnancy and birth defects: a systematic review based on 173 687 malformed cases and 11.7 million controls. Hum. Reprod. Update.

[bib0070] Hare J., Osmond A., Yang Y., Wills G., Weal M., De Roure D. (2009). LifeGuide: a platform for performing web-based behavioural interventions. WebSci’09: Society On-Line.

[bib0075] Health and Social Care Information Centre (2012). Infant Feeding Survey 2010 – Chapter 1: Dietary supplements, smoking and drinking during pregnancy. http://www.hscic.gov.uk/catalogue/PUB08694.

[bib0080] Health and Social Care Information Centre (2013). Statistics on NHS Stop Smoking Services: England, April 2012–March 2013: Report. http://www.hscic.gov.uk/catalogue/PUB12228.

[bib0085] Heatherton T.F., Kozlowski L.T., Frecker R.C., Fagerstrom K.-O. (1991). The Fagerström Test for Nicotine Dependence: a revision of the Fagerstrom Tolerance Questionnaire. Br. J. Addict..

[bib0245] Heil S.H., Scotta T.L., Higginsa S.T. (2009). An overview of principles of effective treatment of substance use disorders and their potential application to pregnant cigarette smokers. Drug Alcohol Depend.

[bib0090] Jones S., Tyler E., Brassey J., De Souza S., Reilly R., Paranjothy S. (2012). Smoking in pregnancy: briefing paper reproductive and early years pathfinder project. http://www.wales.nhs.uk/sitesplus/888/page/64307.

[bib0095] Kozlowski L.T., Porter C.Q., Orleans C.T., Pope M.A., Heatherton T. (1994). Predicting smoking cessation with self-reported measures of nicotine dependence: FTQ, FTND, and HSI. Drug Alcohol Depend..

[bib0100] Kreuter M.W., Skinner C.S. (2000). Tailoring: what's in a name?. Health Educ. Res..

[bib0105] Lie M.L., Hayes L., Lewis-Barned N.J., May C., White M., Bell R. (2013). Preventing type 2 diabetes after gestational diabetes: women's experiences and implications for diabetes prevention interventions. Diabet. Med..

[bib0110] McEwen A., Hajek P., McRobbie H., West R. (2008). Manual of Smoking Cessation.

[bib0115] Meis T., Gaie M., Pingree S., Boberg E.W., Patten C.A., Offord K.P., Berry K.L., Gustafson D.H. (2002). Development of a tailored, Internet-based smoking cessation intervention for adolescents. J. Comput. Mediat. Commun..

[bib0120] Michie S., Brown J., Geraghty A.W., Miller S., Yardley L., Gardner B., Shahab L., McEwen A., Stapleton J.A., West R. (2012). Development of StopAdvisor: a theory-based interactive internet-based smoking cessation intervention. Transl. Behav. Med..

[bib0125] Michie S., Brown J., Geraghty A.W.A., Miller S., Yardley L., Gardner B., Shahab L., Stapleton J.A., West R. (2013). A randomised controlled trial of a theory-based interactive internet-based smoking cessation intervention (‘StopAdvisor’): study protocol. J. Smok. Cessat..

[bib0130] Murray R.L., Bauld L., Hackshaw L.E., McNeill A. (2009). Improving access to smoking cessation services for disadvantaged groups: a systematic review. J. Public Health (Oxf.).

[bib0135] Naughton F., Jamison J., Sutton S. (2013). Attitudes towards SMS text message smoking cessation support: a qualitative study of pregnant smokers. Health Educ. Res..

[bib0140] Naughton F., Prevost A.T., Gilbert H., Sutton S. (2012). Randomized controlled trial evaluation of a tailored leaflet and SMS text message self-help intervention for pregnant smokers (MiQuit). Nicotine Tob. Res..

[bib0145] Naughton F., Prevost A.T., Sutton S. (2008). Self-help smoking cessation interventions in pregnancy: a systematic review and meta-analysis. Addiction.

[bib0150] Park E.R., Quinn V.P., Chang Y., Regan S., Loudin B., Cummins S., Perry K., Rigotti N.A. (2007). Recruiting pregnant smokers into a clinical trial: using a network-model managed care organization versus community-based practices. Prev. Med..

[bib0155] Pollak K.I., Lyna P., Bilheimer A., Farrell D., Gao X., Swamy G.K., Fish L.J. (2013). A pilot study testing SMS text delivered scheduled gradual reduction to pregnant smokers. Nicotine Tob. Res..

[bib0160] Pollak K.I., Oncken C.A., Lipkus I.M., Peterson B.L., Swamy G.K., Pletsch P.K., Lyna P., Brouwer R.J., Fish L.J., Myers E.R. (2006). Challenges and solutions for recruiting pregnant smokers into a nicotine replacement therapy trial. Nicotine Tob. Res..

[bib0165] Raupach T., West R., Brown J. (2013). The most successful method for failing to quit smoking is unassisted cessation. Nicotine Tob. Res..

[bib0170] Rimer B.K., Orleans C.T., Fleisher L., Cristinzio S., Resch N., Telepchak J., Keintz M.K. (1994). Does tailoring matter? The impact of a tailored guide on ratings and short-term smoking-related outcomes for older smokers. Health Educ. Res..

[bib0175] Shahab L., Kenyon J. (2013). The Not-a-Puff rule. National Centre for Smoking Cessation Training. http://www.ncsct.co.uk/usr/pub/not_a_puff_rule.pdf.

[bib0180] Shahab L., McEwen A. (2009). Online support for smoking cessation: a systematic review of the literature. Addiction.

[bib0185] Strecher V.J. (1999). Computer-tailored smoking cessation materials: a review and discussion. Patient Educ. Couns..

[bib0190] Swartz L.H., Noell J.W., Schroeder S.W., Ary D.V. (2006). A randomised control study of a fully automated Internet based smoking cessation programme. Tob. Control.

[bib0195] Te Poel F., Bolman C., Reubsaet A., de Vries H. (2009). Efficacy of a single computer-tailored e-mail for smoking cessation: results after 6 months. Health Educ. Res..

[bib0200] Tombor I., Neale J., Shahab L., Ruiz M., West R. (2014). Healthcare Providers’ Views on Digital Smoking Cessation Interventions for Pregnant Women. J. Smok. Cessat..

[bib0205] Tong V.T., Dietz P.M., Farr S.L., D’Angelo D.V., England L.J. (2013). Estimates of smoking before and during pregnancy, and smoking cessation during pregnancy: comparing two population-based data sources. Public Health Rep..

[bib0210] Ussher M., Beard E., Abikoye G., Hajek P., West R. (2013). Urge to smoke over 52 weeks of abstinence. Psychopharmacology (Berl.).

[bib0215] Ussher M., Etter J.F., West R. (2006). Perceived barriers to and benefits of attending a stop smoking course during pregnancy. Patient Educ. Couns..

[bib0220] Ussher M., West R., Hibbs N. (2004). A survey of pregnant smokers’ interest in different types of smoking cessation support. Patient Educ. Couns..

[bib0225] West R., Brown J. (2013). Theory of Addiction.

[bib0230] West R., Hajek P. (2004). Evaluation of the mood and physical symptoms scale (MPSS) to assess cigarette withdrawal. Psychopharmacology (Berl.).

[bib0235] West R., Stapleton J. (2008). Clinical and public health significance of treatments to aid smoking cessation. Eur. Resp. Rev..

[bib0240] Windsor R.A., Cutter G., Morris J., Reese Y., Manzella B., Bartlett E.E., Samuelson C., Spanos D. (1985). The effectiveness of smoking cessation methods for smokers in public health maternity clinics: a randomized trial. Am. J. Public Health.

