# Supplementary material for: Pilot randomized controlled trial of an internet-based smoking cessation intervention for pregnant smokers (‘MumsQuit’)
Source: Drug Alcohol Depend. 2014 Jul 1;140(100):130–6. doi: 10.1016/j.drugalcdep.2014.04.010 (PMC4067748; doi:10.1016/j.drugalcdep.2014.04.010)

**Supplementary materials for the article**

**Pilot randomized controlled trial of an internet-based smoking cessation intervention for pregnant smokers (‘MumsQuit’).**

Aleksandra Herbec^1^, Jamie Brown^1^, Ildiko Tombor^1^, Susan Michie^2,3^, Robert West^1, 3^

^1^ Cancer Research UK Health Behaviour Research Centre, Department of Epidemiology and

Public Health, University College London, 1-19 Torrington Place, London, WC1E 7HB, UK

^2^ Department of Clinical, Educational and Health Psychology, University College London,

1-19 Torrington Place, London, WC1E 7HB, UK

^3^ National Centre for Smoking Cessation and Training, 1–6 Yarmouth Place, London, W1J 7BU, UK

*Correspondence to*: Aleksandra Herbec, Health Behaviour Research Centre, Rm 215, Department of Epidemiology and Public Health, University College London, 1-19 Torrington Place, London WC1E 7HB, UK. E-mail: aaherbec@gmail.com

This material supplements but does not replace the content of the peer-reviewed paper published in Drug and Alcohol Dependence.

**Example screenshots of MumsQuit intervention**

**MumsQuit pre-quit sessions:**


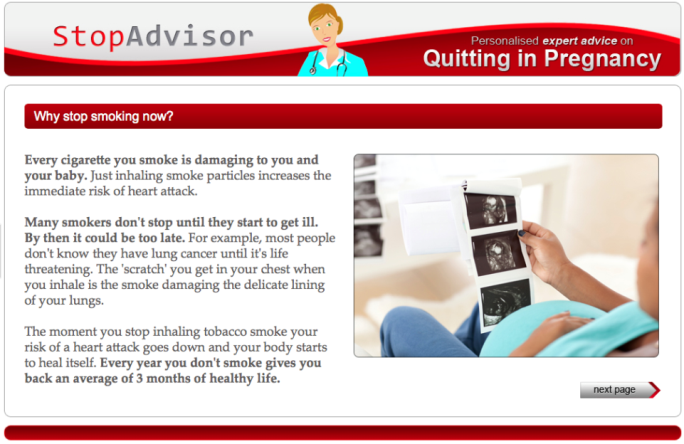

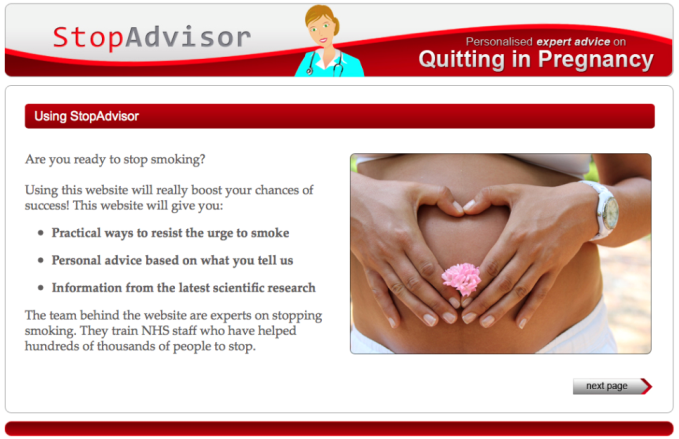


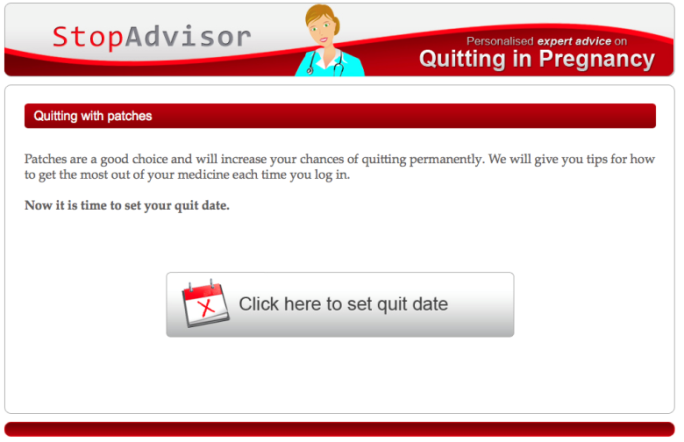

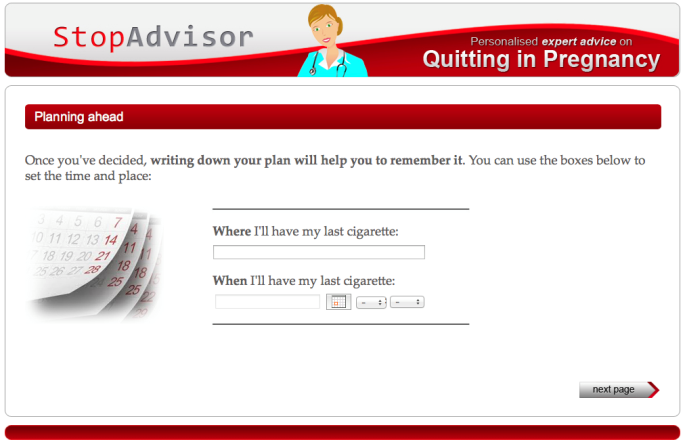


**MumsQuit post-quit sessions:**


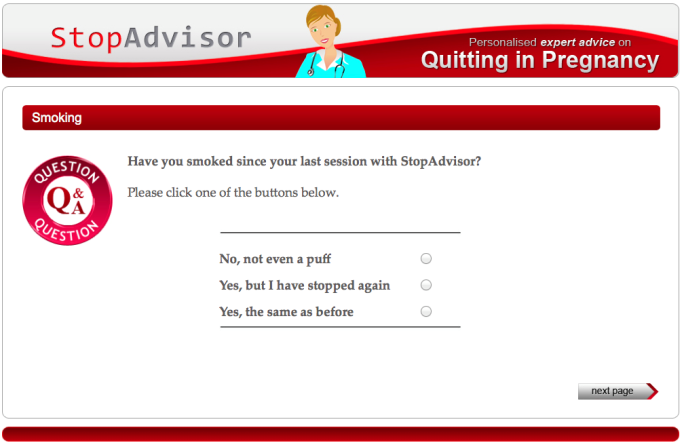

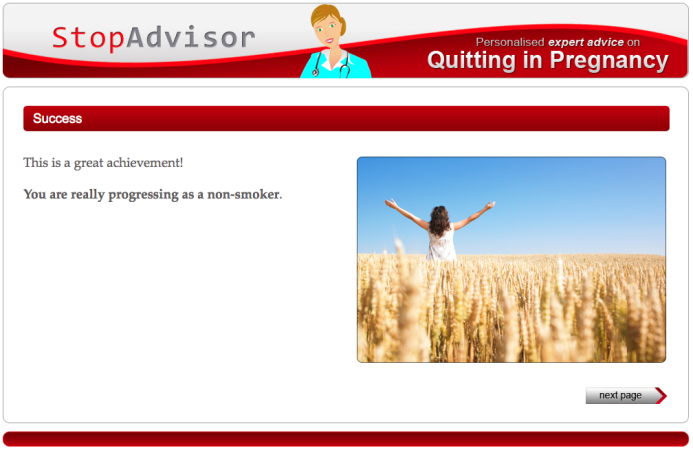


**Control Condition:**


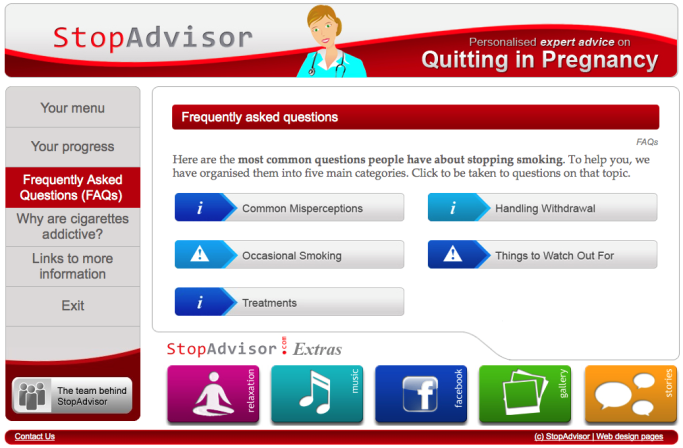

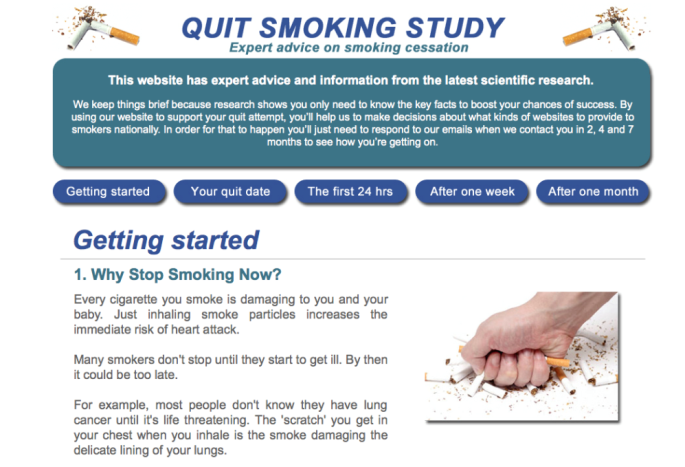

Supplement: Supplementary file 1 [file mmc1.doc]
